# RNA-binding protein HuR regulates the transition of septic AKI to CKD by modulating CD147

**DOI:** 10.1042/CS20241756

**Published:** 2025-01-15

**Authors:** Simeng Liu, Renfei Luo, Davey Li, Anna Tang, Yuli Qiu, Ryan P. Sherrier, Jeffrey Aube, Xiaoqing Wu, Liang Xu, Yufeng Huang

**Affiliations:** 1 Division of Nephrology & Hypertension, Department of Internal Medicine, University of Utah Health Science, Salt Lake City, UT, USA; 2 Division of Nephrology, Department of Internal Medicine, Nanjing Medical University Jiangsu Province Hospital, Nanjing, China; 3 Department of Chemistry, Eshelman School of Pharmacy, University of North Carolina, Chapel Hill, NC, USA; 4 Department of Chemical Biology and Medical Chemistry, Eshelman School of Pharmacy, University of North Carolina, Chapel Hill, NC, USA; 5 Department of Molecular Biosciences, University of Kansas, Lawrence, KS, USA

**Keywords:** chronic kidney disease, HuR inhibitor, LPS, renal inflammation, RNA-binding protein

## Abstract

Septic acute kidney injury (AKI) is an important risk factor for developing chronic kidney disease (CKD). Hu antigen R (HuR) is recognized as a crucial modulator in inflammation. We hypothesized that elevated HuR contributes to the transition from septic AKI to CKD by promoting persistent inflammation and fibrosis, and inhibition of HuR may reverse septic kidney injury. Mice subjected to lipopolysaccharide (LPS) injections every other day were concurrently treated without or with either KH39 or niclosamide (NCS) for 7 days. Control mice received saline injections. Repeated LPS injections led to a significant increase in HuR expression in the kidneys, which was effectively suppressed by KH39 or NCS treatment. LPS-induced kidney injury was characterized by elevated plasma blood urea nitrogen levels and urinary albuminuria, along with histological signs of inflammatory cell infiltration and fibrosis, as determined by periodic acid–Schiff and Masson’s trichrome staining, and immunofluorescent staining for markers such as *α*-smooth muscle actin, fibronectin, collagen III, and F4/80. Treatment with either KH39 or NCS mitigated these changes observed in LPS-injured kidneys. Additionally, increased expression of CD147, a molecule implicated in inflammatory cell recruitment and tubular injury, was inhibited by KH39 or NCS treatment. These effects on HuR and CD147 expression were further validated *in vitro* in cultured macrophages and tubular cells. This study suggests that HuR elevation in LPS-stimulated macrophages and kidney cells contributes to the progression of septic kidney injury, possibly through HuR-CD147 interactions, underscoring the therapeutic potential of HuR inhibitors for this condition.

## Introduction

Sepsis and septic shock are the leading causes of acute kidney injury (AKI), accounting for more than 50% of AKI cases in critically ill patients [1]. The pathogenesis of AKI in sepsis is multifactorial, involving renal hypoperfusion, parenchymal responses to circulating cytokine storms, microvascular injury, inflammation, and microthrombi [2]. These factors contribute to AKI, resulting in a high hospital mortality rate, especially among senior patients. Additionally, many AKI survivors progress to irreversible kidney injury and even end-stage kidney disease, characterized by ongoing interstitial inflammation and renal fibrogenesis, in the absence of cause-specific treatment [2].

The RNA-binding protein Hu antigen R (HuR), known as embryonic lethal abnormal vision-like protein (ELAVL1), is a ubiquitously expressed post-transcriptional regulator [3]. It binds to adenine- and uridine-rich elements (AREs) located in 3′-untranslated region (3′-UTR) of mRNA in response to various stimuli, facilitating mRNA transport from the nucleus to the cytoplasm and preventing rapid degradation [4]. Notably, most pro-inflammatory transcripts contain conserved or semi-conserved AREs in their 3′-UTR [5]. Both the nuclear transcription of HuR and HuR nucleocytoplasmic transporting can be stimulated by inflammatory signals to stabilize inflammatory mediators [6–9]. This HuR/pro-inflammatory circuit likely initiates and maintains the inflammatory phenotype seen in tissue inflammation.

In fact, abnormal elevation of HuR has been observed in kidney diseases, including diabetic nephropathy [10,11], hypertension-related nephropathy [12,13], glomerulonephritis [14], and ischemic kidney injury [15]. Recent studies, including our own, have shown that HuR plays a key role in the progression of chronic kidney disease (CKD) and cardiovascular disease (CVD) by upregulating inflammation and mediating tissue fibrosis [16–18]. We have also discovered potent and specific HuR inhibitors, such as KH3 and KH39 (code: compound 1 c), which disrupt the HuR-ARE interaction [19–21]. Testing these inhibitors for the treatment of CKD is ongoing. Thus, we hypothesized that HuR plays a prominent role in the sepsis-associated kidney injury by promoting persistent inflammation and fibrosis, and that inhibition of HuR could rescue septic kidney injury.

While new specific HuR inhibitors are undergoing continuous preclinical development, several FDA-approved anthelminthic drugs, such as pyrvinium pamoate (PP), have been reported to inhibit HuR by preventing its nucleocytoplasmic accumulation (DOI: 10.18632/oncotarget.9932). We recently identified that niclosamide (NCS), another FDA-approved anthelminthic drug, also inhibits HuR [22,23]. Importantly, NCS is more tolerable and safer than PP *in vivo*. Therefore, we included NCS in this study. Validating the effect of NCS on HuR inhibition and its impact on inflammation and fibrogenesis could enable a rapid transformative approach to treat and reverse septic kidney injury.

Lipopolysaccharide (LPS) injection-induced AKI model has been extensively used to mimic septic AKI in patients [24]. Especially, repeated administration of LPS to mice has led to persistent renal interstitial inflammation and fibrosis [25]. We used a modified version of this repeated LPS injection-induced septic kidney injury model in mice to investigate whether the HuR/pro-inflammatory circuit contributes to the transition from septic AKI to CKD, while also exploring the underlying molecular mechanisms and the therapeutic potential of HuR inhibition for septic kidney injury.

## Materials and methods

### Study 1. *In vivo* studies of changes and inhibition of RNA-binding protein HuR in sustained administration of LPS-induced injury and fibrosis in a mouse model

#### Animals and treatment

Male C57BL/6 mice, aged 10–12 weeks, were obtained from the Jackson Laboratory (Bar Harbor, ME, USA) and used for the induction of kidney injury. Fifteen male mice received initial intraperitoneal (i.p.) injection of LPS (*Escherichia coli, serotype O55:B5*, Sigma) dissolved in sterile saline at the dose of 5 mg/kg body weight (BW) on day 1. This dose was determined in our pilot study, where male mice received different single doses (5.0, 7.5, and 10 mg/kg BW) of LPS (*E. coli, serotype O55:B5*). A low dose of LPS (5 mg/kg BW) for 48 hours induced elevated circulating cytokine release, plasma blood urea nitrogen (BUN) levels, and locally increased renal inflammatory factor expression without causing mouse death. LPS-injected male mice were then randomly assigned to three groups (*n* = 5 per group): one group received no additional treatment, while the other two groups were treated with either KH39 (50 mg/kg BW) or niclosamide (NCS, 10 mg/kg BW) i.p. daily for seven days. KH39 was dissolved in 0.9% NaCl solution with 5% DMSO and 5% Tween-80, and the effective dose of KH39 had been previously determined [21]. NCS was dissolved in PBS with 5% ethanol and 5% Tween-80 as described previously [23]. All LPS-injected mice continued to receive LPS injection at the same dose (5 mg/kg BW) i.p. every other day, for a total of four doses. Normal male mice injected with saline severed as controls (*n* = 5). All mice were housed in standard cages with a 12-hour light/dark cycle, given water and normal diet ad libitum. On day 6, all mice were placed in the metabolic cages individually, and 24-hour urine samples were collected from day 6 to day 7.

This study was initially conducted in male mice, as the optimal dose of LPS-induced kidney injury and the therapeutic doses of NCS and KH39 were first determined in males [21,23]. While LPS affects both male and female mice, the doses of LPS, NCS, or KH39 required for females may differ. Future studies will include testing in female mice.

#### Euthanasia

All mice were euthanized under isoflurane anesthesia on day 7. Blood and kidney samples were harvested on day 7 as described previously [17].

#### Determination of renal function and albuminuria

Plasma BUN concentrations were measured by using the QuantiChromTM urea assay kit (BioAssay System, Hayward, CA, USA). Urinary creatinine (Cr) levels were measured by using a creatinine liquicolor kit (no. 0420250, Stanbio Laboratory). Urinary albumin levels were determined by a murine microalbuminuria ELISA kit (No. 1011, Exocell), and urinary albumin/creatinine (A/C) ratio was further calculated.

#### Histological examination

Four-micrometer sections of paraffin-embedded kidney tissues were stained with periodic acid-Schiff (PAS) and Masson’s Trichrome (TRI) by the histology core facility at the University of Utah. Ten random fields from each kidney section were analyzed under ×200 magnification. The deposition of collagen, stained blue, was quantified using imageJ and presented as a percentage of the total analyzed area in a blinded fashion. The average blue staining score for 5 mice in each group was calculated and graphed. This method differs from the one described previously [17].

Immunofluorescent staining (IF) for HuR and CD147 was performed on paraffin-embedded kidney tissues as described previously [16,17]. The monoclonal mouse anti-HuR IgG and mouse anti-CD147(EMMPRIN) IgG (Santa Cruz Biotechnology, Inc., Santa Cruz, CA, USA) served as the primary antibodies, and Alex Fluor Plus 594-conjugated goat anti-mouse IgG (H + L) (Invitrogen, Carlsbad, CA, USA) served as the secondary antibody. At the same time, fluorescein isothiocyanate (FITC)-conjugated wheat germ agglutinin (WGA) (ThermoFisher Scientific, USA) was used to counterstain the glomeruli and tubules to define the location of HuR and CD147 in the kidney. DAPI-Fluoromount-G (SouthernBiotech, Birmingham, AL, USA) was used to stain the nuclei DNA. Control slides treated with antibody diluent instead of primary antibodies showed no staining.

Immunofluorescent staining for *α*-smooth muscle action (*α*-SMA), fibronectin (FN) or type III collagen (Col-III) or F4/80 + positive cells were performed and quantified on paraffin-embedded kidney sections as described previously [26–28]. Either rabbit anti-human FN IgG or goat anti-human type III collagen (Southern Biotechnology Associates, Birmingham, AL) or rat anti-mouse F4/80 IgG (Bio-Rad Laboratories, Inc., Hercules, CA, USA) served as the primary antibody, respectively. FITC-conjugated goat anti-rabbit IgG, Rhodmain-Red^X^-conjugated donkey anti-goat IgG or CyTM3-conjugated goat anti-rat IgG (Jackson ImmunoResearch Laboratories Inc., West Grove, PA, USA) were used as the secondary antibody. Control slides treated with antibody diluent instead of primary antibodies showed no staining. For immunostaining of *α*-SMA, FITC-conjugated mouse anti-*α*-SMA antibody was used directly. Ten random fields from each kidney section were analysed under ×200 magnification. Digital morphometric measurement of *α*-SMA, or FN, or Col-III positive staining, or F4/80 + positive cells was quantified as the percentage of staining positive area occupied in the total analysed area using ImageJ (National Institutes of Health, Bethesda, MD, USA). The average positive staining score for five mice in each group was calculated and graphed.

#### Western blot measurement

Kidney protein from each animal of each group was isolated and then immunoblotted on immobilon-P transfer membranes (ThermoFisher Scientific) as described previously [16,28]. Proteins for HuR, CD147, *α*-SMA, fibronectin (FN), and GAPDH were assessed on the blots. The antibody information and analysis of the immunostaining bands were described previously [16,26,28–30]. All blots were run at least two times.

### Study 2. *In vitro* studies on the effects of KH39 and NCS on cellular HuR and CD147 expression following LPS stimulation

#### Cell culture and reagents

Mouse macrophage cell line, RAW264.7 and mouse proximal kidney tubular epithelium cells (TCMK-1) were purchased from American Type Culture Collection (ATCC) and cultured in DMEM medium supplemented with 10% fetal bovine serum (FBS), 100 μg/ml streptomycin and 100 U/ml penicillin (all from Gibco, Thermo-Fisher Scientific, Waltham, MA, USA) at 37°C in a 5% CO_2_ incubator. Sub-confluent cells seeded on six-well plates were made quiescent in serum-free DMEM medium for 24 hours before experimental studies. KH39 and NCS were dissolved in DMSO at 20 mM as stock solutions for *in vitro* assays. LPS (*E. coli, serotype O55:B5*, Sigma) was dissolved in sterile saline. All cellular treatments were carried out in duplicates in separate wells and repeated three times.

#### Effect of LPS on cellular HuR expression and nucleocytoplasmic translocation

The quiescent cells were first treated with LPS at different doses for 24 hours, and cell viability was then measured to determine the optimal culture dose of LPS. Second, the quiescent cells were treated with an optimal dose of LPS (5 µg/ml for RAW264.7 cells and 10 µg/ml for TCMK-1 cells) and then collected at different time points after treatment for HuR immunocytofluorescent staining as described above. Incubation for 24 hours or 48 hours was chosen as optimal for LPS to induce nucleocytoplasmic translocation of HuR with or without HuR inhibitor in cultured RAW264.7 (24 hours) and TCMK-1 (48 hours) cells, respectively. Third, the dose of HuR inhibitor, KH39 or NCS, was optimized in cultured cells before intervention. Finally, the quiescent cells were incubated in serum-free medium alone or serum-free medium with LPS, LPS plus KH39 (2 μM), or LPS plus NCS (1 μM). HuR inhibitor-treated cells were preincubated with KH39 or NCS for 30 minutes before adding LPS. Cells were harvested at the indicated times for HuR staining (described above) and measurement of cytoplasmic/nuclear/total cellular HuR protein production by Western blotting as described above. Cytoplasmic and nuclear proteins were isolated separately by using the Thermo-Scientific™ NE-PER™ Nuclear and Cytoplasmic Extraction Reagent kit (ThermoFisher Scientific) as described previously [17]. The band intensities were measured using ImageJ and normalized to *ß*-actin (cytoplasmic) or histone 3 (nuclear).

#### Effect of KH39 and NCS on LPS-induced CD147 expression in RAW264.7 cells and TCMK-1 cells

The quiescent RAW264.7 cells, or TCMK-1 cells were treated with LPS with or without KH39 or NCS at the indicated concentration and incubation time. Cells were then harvested for measurement of total protein production of CD147 by Western blotting as described previously [22]. The band intensities were measured using ImageJ and normalized to *ß*-actin.

### Statistical analysis

All data are expressed as mean ± SD. Software power for sample size calculation (www.clincalc.com) was used for the *in vivo* study, based on the results of renal tubular injury score in a pilot study. Each group contains five mice, and the study has at least 95% power to detect differences larger than 2.2 units of standard deviation between treated and untreated groups. Statistical analyses of differences among the groups were performed by one-way ANOVA, and subsequent Student-Newman-Keuls or Dunnett’s testing for multiple comparisons. Comparisons with *P* < 0.05 were considered significantly different.

## Results

### Elevated HuR is observed in LPS-injured kidneys in a mouse model

**Figure 1:**
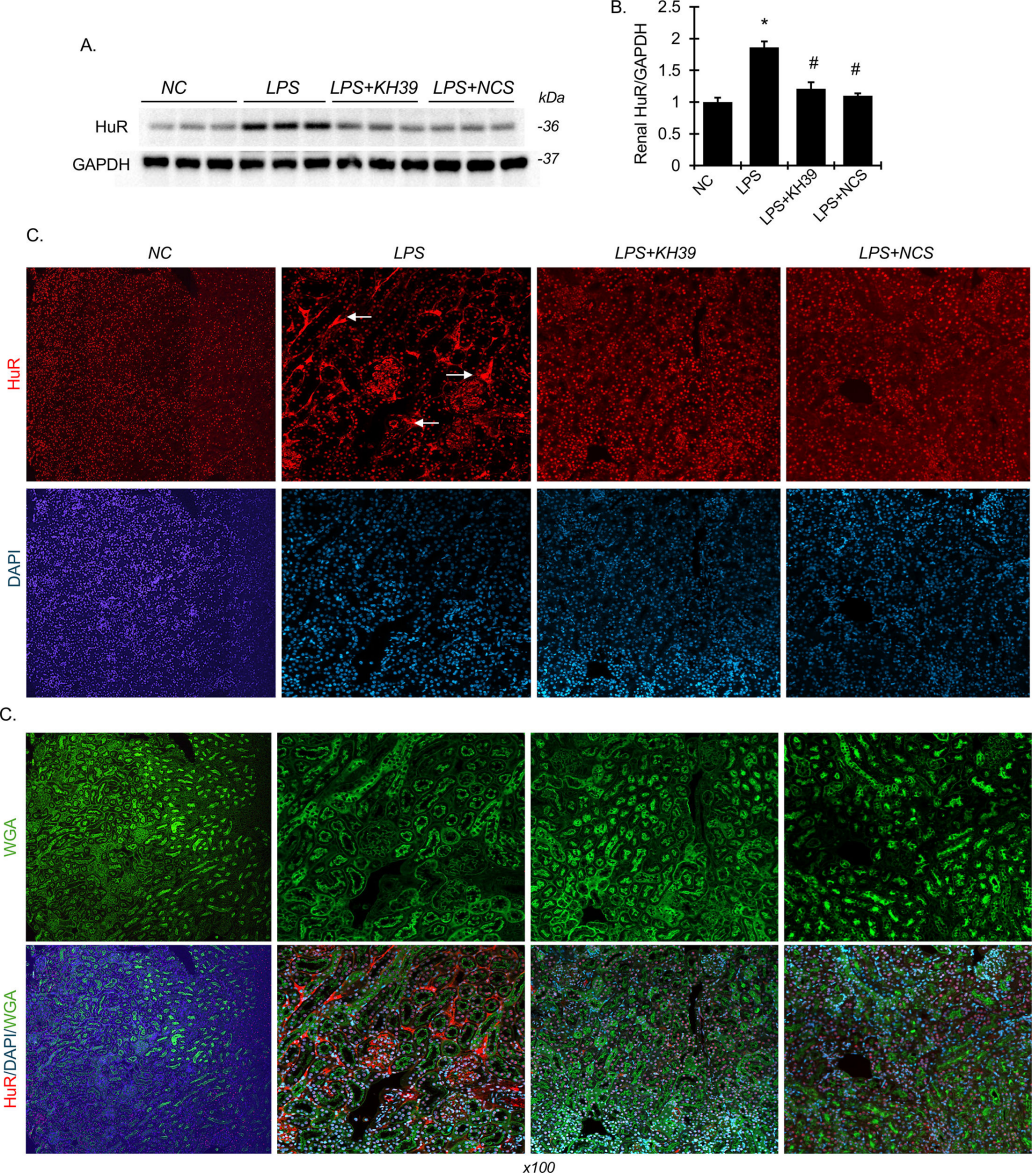
LPS increases renal HuR protein production and staining in the mouse kidney. (**A)** Representative Western blots illustrate the protein expression of HuR and GAPDH in the whole kidney tissue. (**B)** Quantification of the band density is shown on the right of Western blot. Protein values are expressed relative to normal control, which was set to unity (*n* = 5/each group). (**C)** Representative photomicrographs of renal immunofluorescent staining for HuR (red) at ×100 magnification are shown from normal mice (NC), LPS-injected mice without treatment (LPS), and LPS-injected mice treated with either KH39 (LPS+KH39) or NCS (LPS+NCS) (*n* = 5/each group). Possible cytoplasmic staining for HuR is indicated by arrows in LPS-injured kidneys. The nuclear control staining with DAPI and kidney structure staining with wheat germ agglutinin (WGA) (green) were shown below the HuR. *P < 0.05, vs NC; #*P*<0.05, vs LPS.

As shown in Figure 1A and B, we observed that HuR protein was weakly expressed in normal kidney tissue in mice (NC). In contrast, significantly increased HuR protein expression was observed in diseased kidneys induced by repeated LPS injections, which was inhibited by HuR inhibitors KH39 and NCS. Immunofluorescent (IF) staining for HuR confirmed increased staining density for HuR and possible nucleocytoplasmic translocation of HuR (stained red) in tubule and tubulointerstitial cells, as well as some glomerular cells at the site of injury (Figure 1C-LPS, arrows pointed). This indicates increased HuR expression. Consistently, the enhanced HuR staining was not seen in normal mice and was barely detectable in KH39- or NCS-treated LPS-injured mice. These results suggest that renal HuR production was increased in LPS-injured kidneys. Additionally, these findings confirm the inhibitory ability of NCS on LPS-induced elevated HuR production in the kidneys.

### Inhibition of HuR improves renal function and reduces albuminuria and renal inflammation and fibrosis in a mouse mode of LPS-induced kidney disease

**Figure 2:**
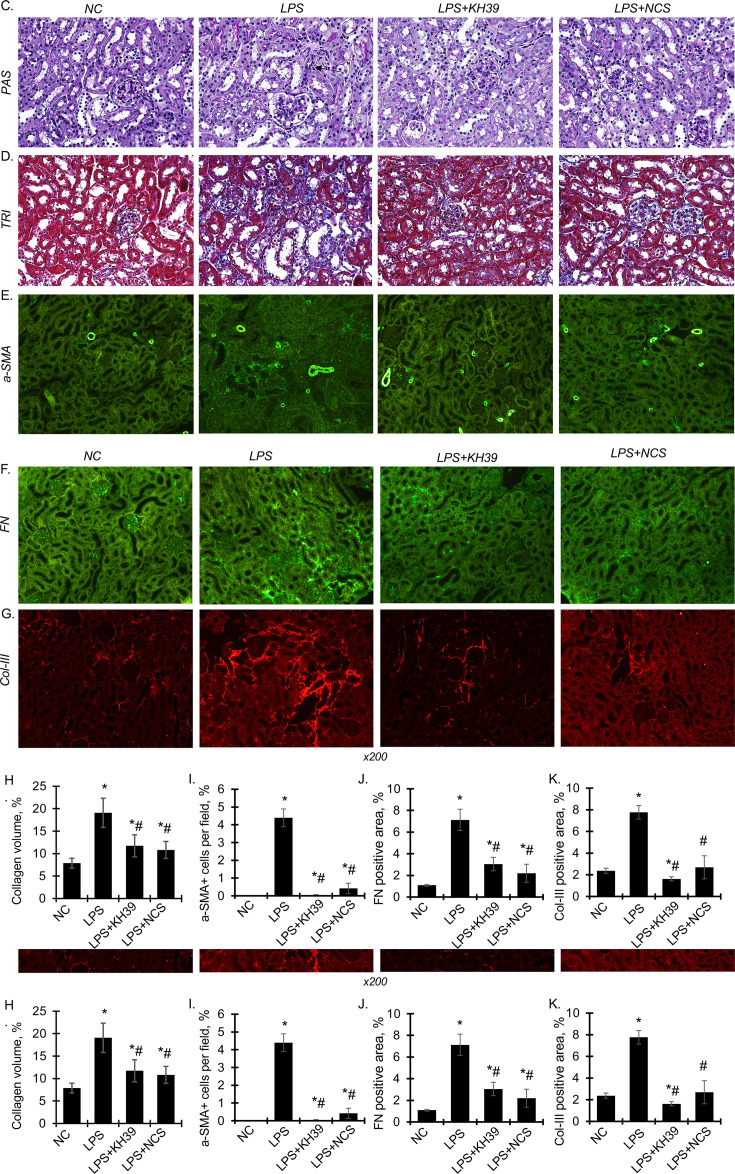
Inhibition of HuR ameliorates LPS-induced elevated plasma BUN levels, urinary albumin/creatinine ratio (A/C), kidney injury and fibrosis in mice. (**A)** Serum BUN levels, *n* = 5/each group. (**B)** Urinary A/C levels. (**C, D)** Representative microscopic images showing PAS staining (**C**) and Masson’s Trichrome staining (**D**) of kidney sections used to detect tubular injury, inflammation, and collagen deposition (stained blue). Magnification, ×200. (**E–G)**. Kidney sections from normal mice (NC) and LPS injected mice without (LPS) and with KH39 (LPS+KH39) or NCS (LPS+NCS) treatment were stained with a-SMA (green) (**E**), fibronectin (FN) (green) (**F**) and type III collagen (Col-III) (**G**). Magnification, ×200. (**H–K)**. The graphs summarize the results of collagen (**H**), a-SMA (**I**), FN (**J**), and Col-III (**K**) deposition quantified using ImageJ and presented as a percentage of the total analysed area. *P < 0.05 versus NC; #*P* < 0.05 versus LPS. *n* = 5/each group.

All mice survived the experiment period. As shown in Figure 2A and B, repeated LPS injection only for seven days induced continued kidney damage in mice, including increased plasma BUN levels and urinary albumin/creatinine (A/C) ratio. Histologically, LPS-injured kidneys showed accumulative inflammatory cell infiltration and tubulointerstitial collagen deposition, as determined by PAS staining and Masson’s trichrome (TRI) staining (Figure 2C and D). The quantitative analysis of renal collagen deposition is shown in Figure 2H. These results suggest that repeated LPS injections for seven days can induce significant kidney injury in mice, which may initiate the transition from AKI to CKD. In contrast, mice treated with either KH39 or NCS showed a significant reduction in BUN levels and albuminuria compared to untreated LPS-injured mice (Figure 2A and B), as well as significantly less tubular injury, inflammation, and tubulointerstitial fibrosis (Figure 2A–D and G).

**Figure 3:**
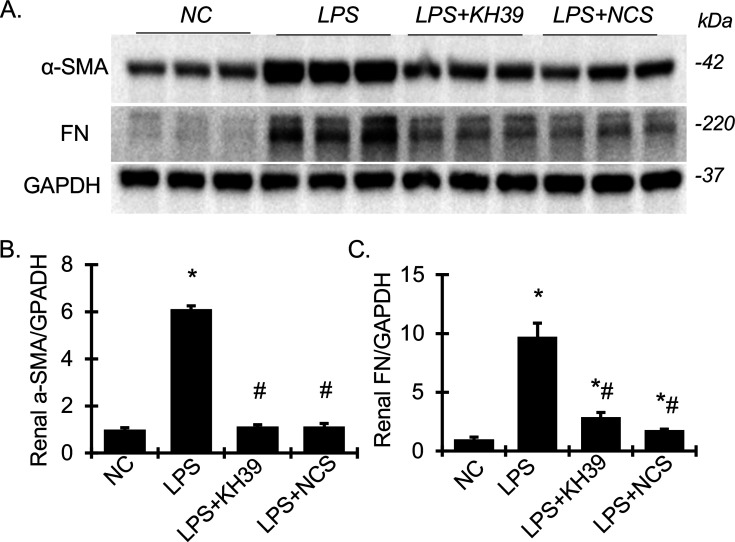
Inhibition of HuR reduces renal protein levels of fibrotic markers following LPS injection. **(A)** Western blots of a-SMA, FN, and GAPDH from normal mouse kidneys and LPS-injured kidneys of untreated and treated mice. Molecular weight is labelled on the right. (**B, C)**. The graphs summarize the results of band density measurements for a-SMA (**B**) and FN (**C**) in the kidney following LPS injury. *n* = 5/each group. *P < 0.05, versus normal control mice (NC); #*P* < 0.05, versus LPS-injected mice without treatment (LPS). LPS+KH39 or LPS+NCS: LPS-injected mice treated with KH39 or NCS.

The immunofluorescent-stained kidneys for *α*-smooth muscle actin (*α*-SMA), fibronectin (FN), and type III collagen (Col-III) (Figure 2E–G) and their semi-quantitative analyses in Figure 2I–K, confirmed that overexpression of *α*-SMA was observed not only in vascular smooth muscle cells (VSMCs) but also in LPS-injured kidney tubular and/or tubulointerstitial or peritubular cells. The positive IF staining for FN and Col-III on tubular basement membrane and tubulointerstitial area was dramatically increased in LPS-injured kidneys. However, the IF staining for these fibrotic markers was markedly reduced when LPS-injured mice were treated with either KH39 or NCS. This observation was further supported by Western blot analyses (Figure 3), which showed a striking elevation in renal protein levels of *α*-SMA and FN in LPS-injured kidneys compared to the normal controls. This elevation was abrogated in LPS-injured mice treated with KH39 or NCS. These data together indicate that treatment with a HuR inhibitor protects the kidney from LPS-induced tubular fibrosis.

**Figure 4:**
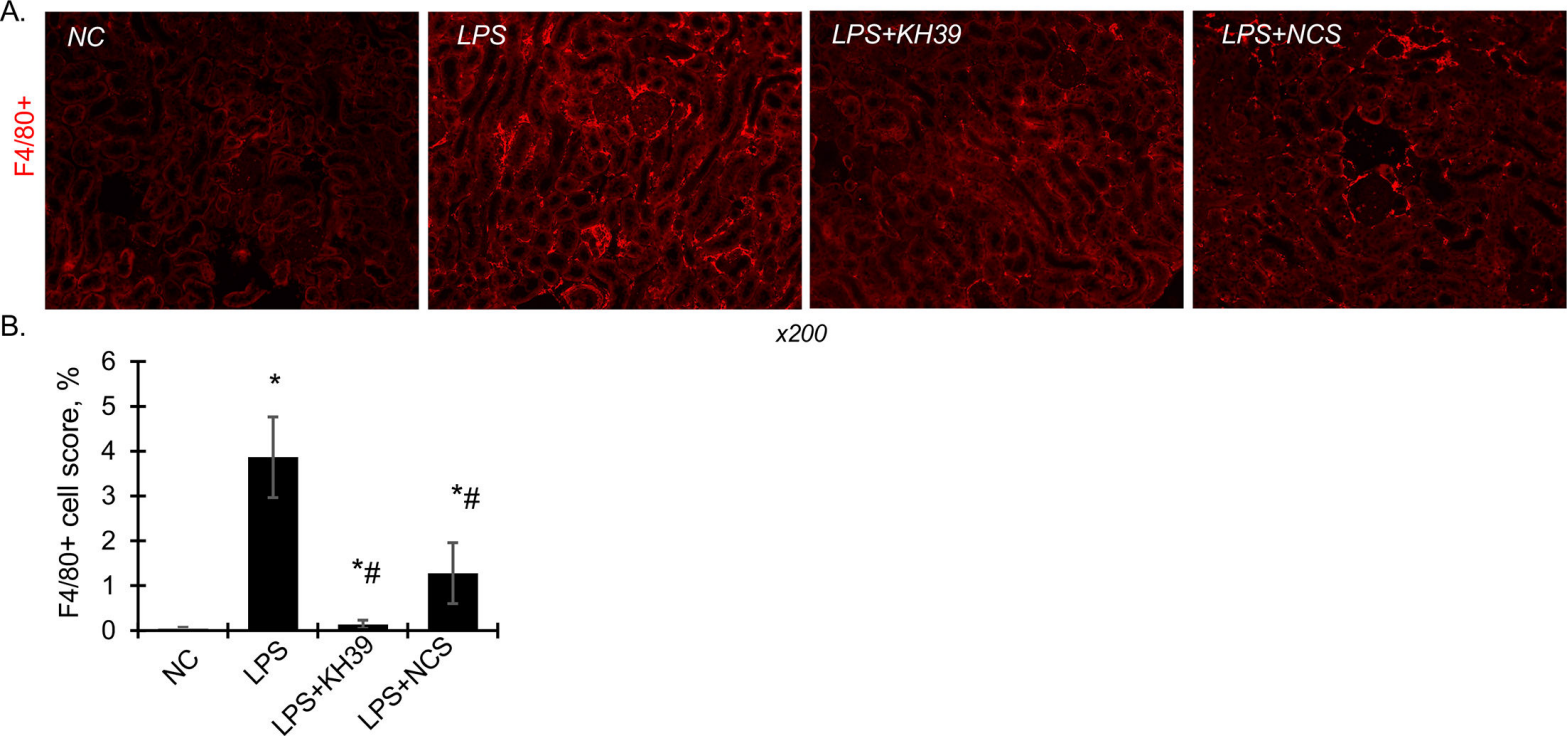
Inhibition of HuR reduces macrophage infiltration in the kidney after LPS injection. **(A)** Kidney sections from normal mice (NC), untreated and KH39 or NCS-treated mice following LPS injection were stained with F4/80 (red). Magnification, ×200. (**B)** The graphs summarize the results of F4/80^+^ cells, quantified using ImageJ and presented a percentage of the total analysed area. *n* = 5/each group. *P < 0.05 versus NC; #*P* < 0.05 versus LPS.

The F4/80 antibody is known to label macrophages. LPS-injured mice had a substantial increase in the absolute number of F4/80 + cells, mainly in the tubulointerstitial area, while F4/80 + cells were sparse in renal vessels in normal control kidneys, indicating an accumulation of macrophages and inflammation in damaged kidneys (Figure 4). However, the number of F4/80 + cells was largely reduced after treatment with KH39 or NCS, nearing the normal levels observed in uninjured kidneys. These results indicate that sepsis-induced kidney inflammation was substantially reduced in mice by KH39 or NCS treatment.

### Inhibition of HuR modulates renal CD147 expression in LPS-injured mice

**Figure 5:**
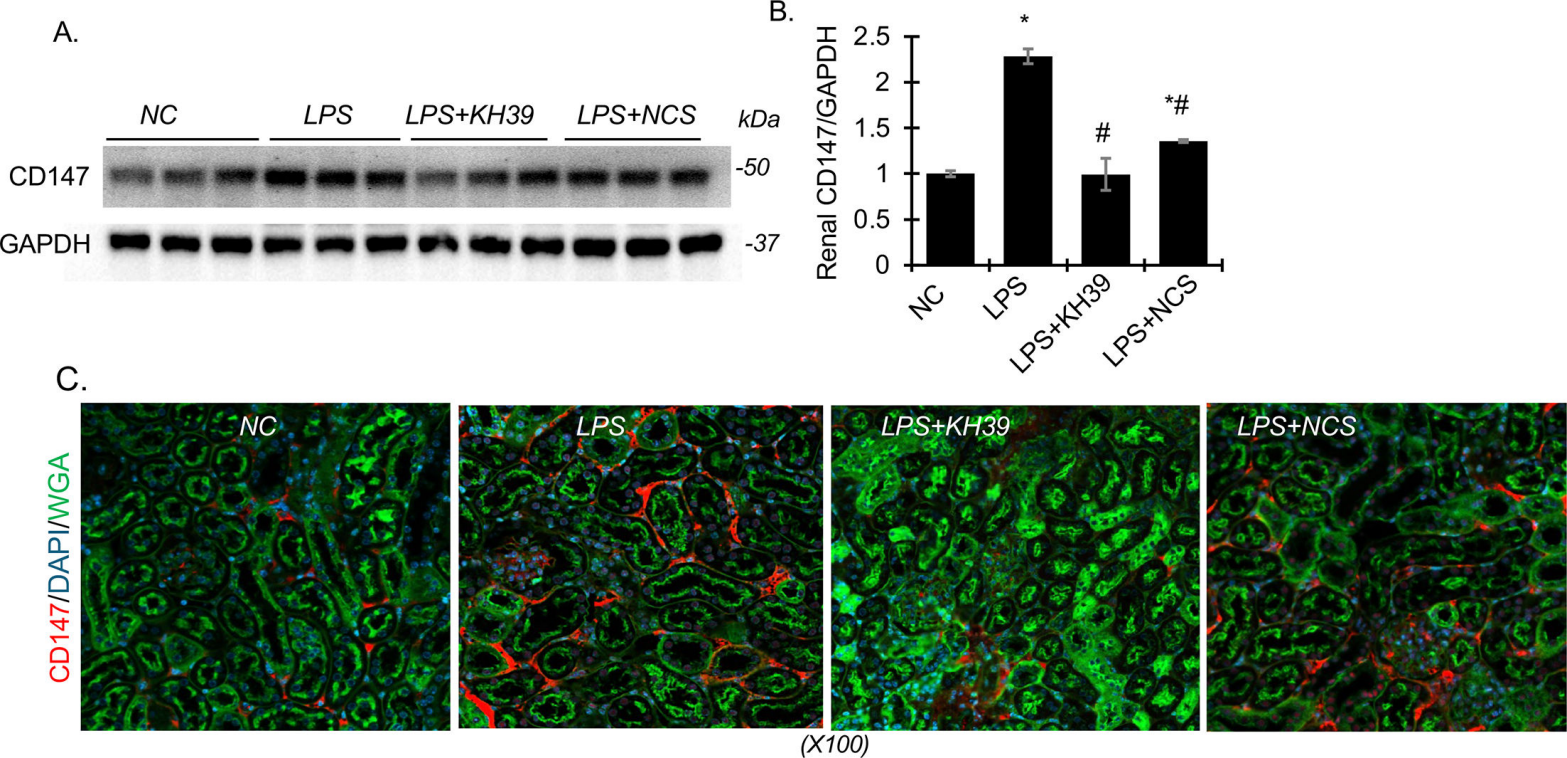
LPS stimulates renal CD147 protein production and immunofluorescent staining in the mouse kidney. (**A)** Representative Western blots illustrate the protein expression of CD147 and GAPDH in the whole kidney tissue. (**B)** Quantification of the band density is shown on the right of Western blot. Protein values are expressed relative to normal control, which was set to unity. *n* = 5/each group. (**C)** Representative photomicrographs of renal immunofluorescent staining for CD147 (red), DAPI (blue) and wheat germ agglutinin (WGA) (green) at ×100 magnification are shown from normal mice (NC), LPS-injected mice without treatment (LPS), and LPS-injected mice treated with either KH39 (LPS+KH39) or NCS (LPS+NCS). *P < 0.05, versus NC; #*P* < 0.05, versus LPS.

CD147, also called extracellular matrix metalloproteinase inducer (EMMPRIN) or basigin, is a glycosylated transmembrane [31]. It includes two forms, highly glycosylated CD147 (HG-CD147, ~50 kDa) and lowly glycosylated CD147 (LG-CD147, ~30 kDa). Interestingly, both forms of CD147 in the kidney increased by 2.28-fold after LPS injury, and this increase was inhibited by either KH39 or NCS (Figure 5A,B). IF staining for CD147, using the same primary mouse-anti CD147 used in the Western blot analysis, confirmed the enhanced staining of CD147 (stained red) in tubular basement membrane and tubulointerstitial cells at the site of injury (Figure 5C-LPS, arrows pointed), compared to normal mice. KH39 or NCS treated LPS-injured mice had much less CD147 staining in the kidneys. From both measurements of CD147, NCS was less effective than KH39 in reducing CD147, which may be related to the different doses of the drugs used.

### LPS directly induces cellular expression and nucleocytoplasmic translocation of HuR in macrophages and renal tubular cells

**Figure 6:**
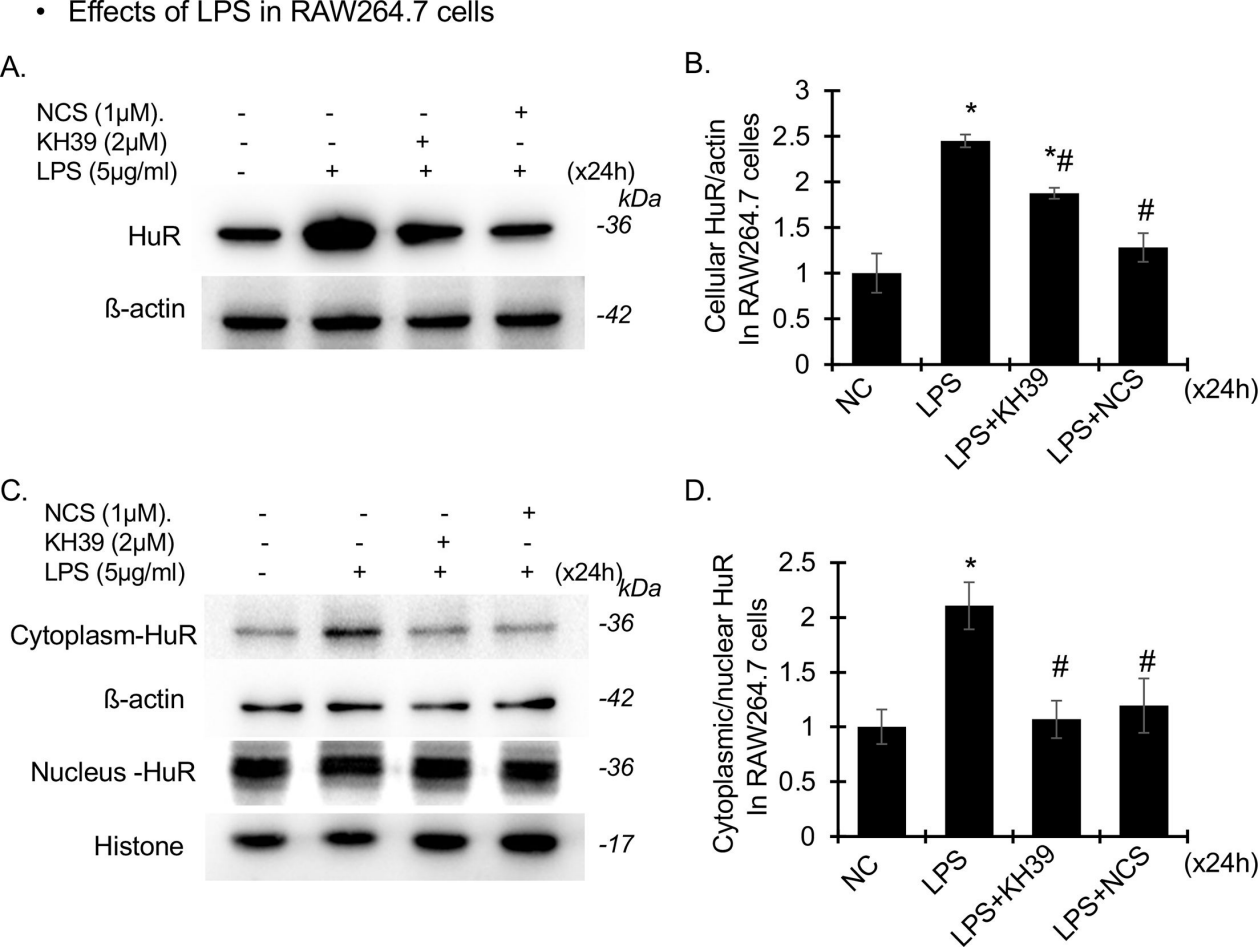
LPS induces the total HuR expression and its nucleocytoplasmic translocation in cultured murine macrophages cells, which is inhibited by KH39 and NCS. (**A)** Representative Western blots illustrate the total cellular protein production of HuR and ß-actin in cultured RAW264.7 cells. (**B)** Quantification of the band density is shown on the right of Western blot. Protein values are expressed relative to normal control cells, which was set to unity. (**C)** Representative Western blots illustrate cytoplasmic and nucleus HuR and ß-actin and histone protein expression in untreated and treated RAW264.7 cells. (**D)** The graphs summarize the results of band density measurements for the ratio of cytoplasmic HuR to nuclear HuR. The ratio values are expressed relative to untreated normal control cells, which were set to unity. Cell treatment was repeated three times. *P < 0.05, versus untreated normal control cells (NC); # *P* < 0.05, versus LPS-treated cells without HuR inhibition (LPS).

**Figure 7:**
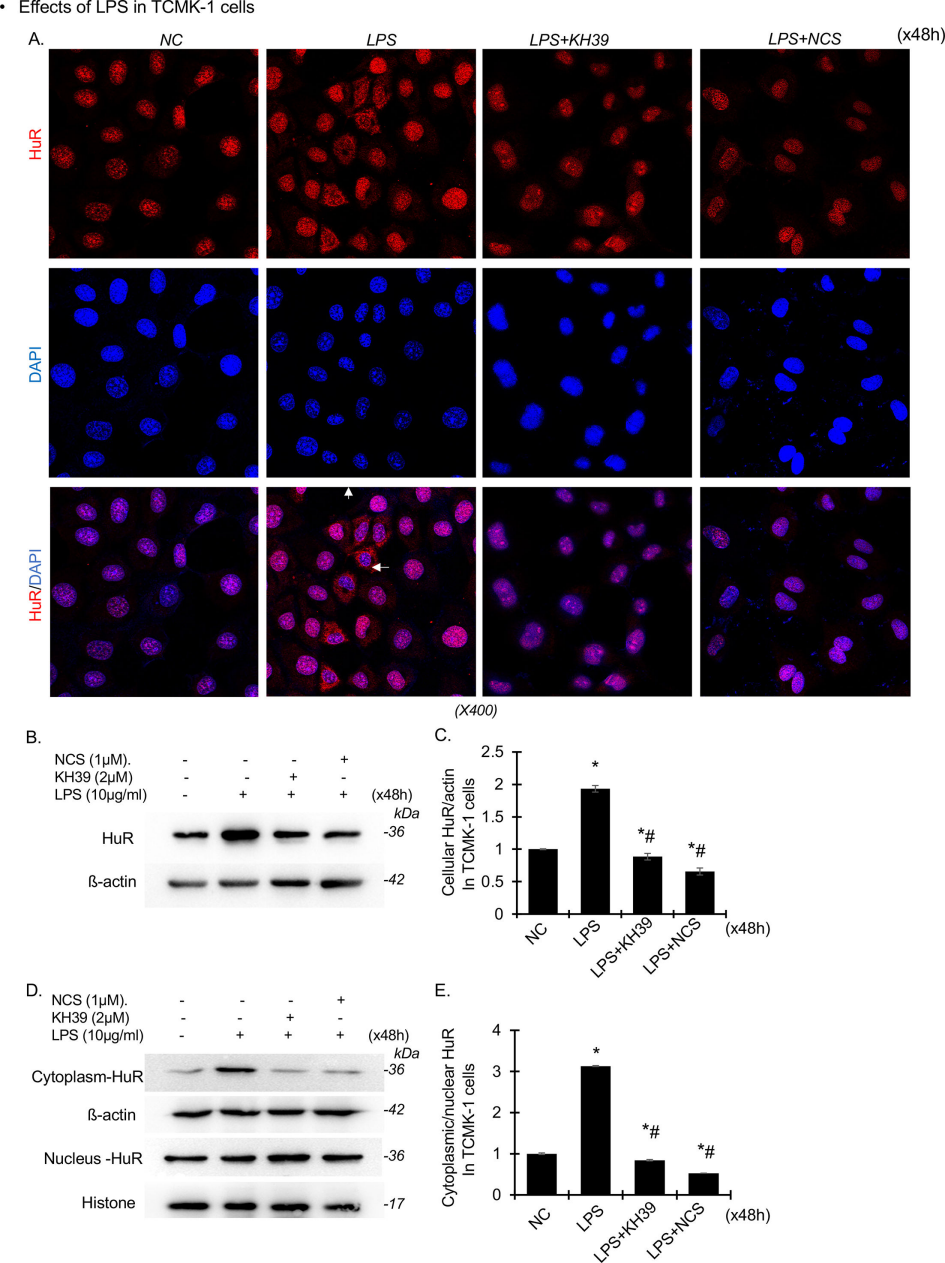
LPS induces the total HuR expression and its nucleocytoplasmic translocation in cultured murine renal tubular cells, which is inhibited by KH39 and NCS. (**A)** Representative photomicrographs of cellular immunofluorescent staining for HuR (red) and the nuclei (blue) at ×400 magnification from untreated TCMK-1 cells (NC), TCMK-1 cells treated with LPS alone (LPS), LPS plus KH39 (LPS+KH39) or LPS plus NCS (LPS+NCS) for 48 hours. Arrows indicate cytoplasmic staining of HuR. (**B)** Representative Western blots illustrate the total cellular protein production of HuR and *β*-actin in cultured TCMK-1 cells. (**C)** Quantification of the band density is shown on the right of Western blot. Protein values are expressed relative to normal control cells, which was set to unity. (**D)** Representative Western blots illustrate cytoplasmic and nuclear HuR and *ß*-actin and histone protein expression in untreated and treated TCMK-1 cells. (**E)** The graphs summarize the results of band density measurements for the ratio of cytoplasmic HuR over nucleus HuR. The ratio values are expressed relative to untreated cells, which were set to unity. Cell treatment was repeated three times. *P < 0.05, versus NC; # *P* < 0.05, versus LPS.

As expected, administration of LPS for 24 hours directly induced total cellular (Figure 6A and B) and cytoplasmic (Figure 6C and D) HuR production levels in cultured macrophages, and these effects were inhibited by KH39 or NCS. Surprisingly, administration of LPS for 48 hours induced cellular HuR density (shown in red staining) and nucleocytoplasmic translocation of HuR (red staining was shown in cytoplasm) in cultured renal tubular cells, and these effects were similarly abrogated by KH39 or NCS treatment (Figure 7A). Western blot assays further confirmed the observation of IF staining for HuR in renal tubular cells. LPS similarly induced total cellular (Figure 7B and C) and cytoplasmic (Figure 7D and E) HuR levels in renal tubular cells, and these effects were inhibited by KH39 or NCS. These results indicate that HuR is increased in both LPS-stimulated macrophages and kidney cells, but macrophages respond to LPS in less time and at lower doses than tubular cells do. In addition, NCS effectively blocks HuR nucleocytoplasmic translocation, acting as a HuR inhibitor.

### Both KH39 and NCS decrease CD147 expression in macrophages and renal tubular cells

**Figure 8:**
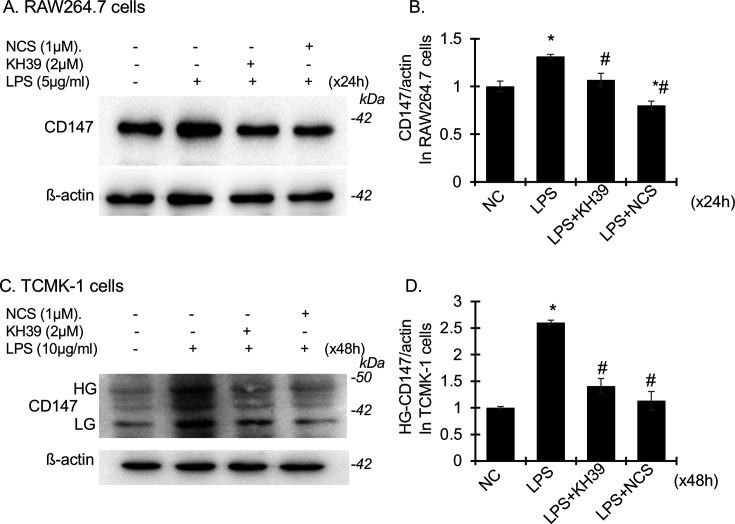
HuR inhibition abrogates LPS-induced cellular expression of CD147 both in cultured murine macrophages and renal tubular cells. (**A and C)** Representative Western blots illustrate CD14 and β-actin protein expression in untreated or treated RAW264.7 cells (**A**) and untreated or treated TCMK-1 cells (**C**). **(B and D)** The graphs summarize the results of band density measurements for CD147 in RAW264.7 cells (**B**) and TCMK-1 cells (**D**), respectively. The protein values are expressed relative to untreated normal cells, which were set to unity. Cell treatment was repeated three times. *P < 0.05, versus untreated normal control cells (NC); # *P* < 0.05, versus LPS-treated cells without HuR inhibition (LPS).

As shown in Figure 8A and B, macrophages mainly expressed LG-CD147, not HG-CD147. LPS stimulation further enhanced LG-CD147 expression in macrophages, which was significantly inhibited by KH39 or NCS treatment. Interestingly, renal tubular cells expressed both HG-CD147 and LG-CD147 after LPS stimulation (Figure 8C and D). LPS-stimulated cells treated with HuR inhibitors (either KH39 or NCS) showed reduced HG-CD147 and LG-CD147 expression levels. Our data further demonstrates that CD147 is a target of HuR, and HuR inhibition downregulates CD147 expression in both LPS-activated macrophages and injured renal tubular cells.

## Discussion

The present study utilized a mouse model induced by repeated low-dose LPS administration over one week, which results in impaired renal function, albuminuria, and persistent renal interstitial inflammation and fibrosis. This model effectively mimics the transition of AKI to CKD in humans, particularly in the context of sepsis, and aligns with observations in endotoxemia-associated kidney injury and fibrogenesis [25]. Notably, we observed a significant increase in HuR expression following LPS stimulation in both inflammatory and kidney cells in *in vivo* and *in vitro* settings. Inhibition of HuR alleviated renal interstitial inflammation and fibrosis, improving renal function and reducing albuminuria. These findings suggest that HuR plays a crucial role in LPS-induced kidney disease, potentially through its downstream target CD147, underscoring the therapeutic potential of HuR inhibitors for septic kidney injury.

The inflammatory effects of LPS are well-documented and involve both circulating immune cells and local organ cells. Upon entering the bloodstream, LPS activates immune cells such as macrophages and monocytes, promoting the release of pro-inflammatory cytokines and chemokines. These mediators amplify the inflammatory response by recruiting additional immune cells to the site of infection or injury. Simultaneously, LPS directly affects local organ cells, including renal epithelial cells, stimulating them to produce inflammatory cytokines and chemokines, exacerbating organ inflammation and tissue damage. LPS-induced vascular endothelial dysfunction further increases vascular permeability, enhancing leukocyte infiltration into tissues. These processes create a complex interplay between circulating immune cells and local organ cells, driving tissue-specific inflammation and injury.

Emerging evidence suggests that LPS upregulates a variety of HuR-bound transcripts in circulating immune cells, which are involved in innate immunity, cytokine activity, and chemotaxis [32]. These transcripts likely contribute to the overproduction of proinflammatory cytokines during critical infections. However, there is limited research on LPS-induced HuR dysfunction in local organ cells, particularly in the kidney.

In this study, we found that LPS stimulates multiple kidney cell types, including glomerular, tubular, and tubulointerstitial cells, to overexpress HuR. Elevated HuR expression in glomeruli, particularly in podocytes, has been linked to podocyte injury and proteinuria [33]. Similarly, increased HuR levels in kidney tubular cells—observed here and in our previous ischemia/reperfusion-injured model [17]—may sensitize these cells to TGFß-induced proinflammatory and profibrotic signaling, promoting tubulointerstitial fibrosis. We also identified that LPS upregulates CD147 expression in renal tubular cells via a HuR-mediated mechanism. Previous studies suggest that elevated tubular CD147 expression promotes profibrotic tubular epithelial differentiation by inducing MMP generation and enhancing proinflammatory responses via STAT3 signaling [34,35]. These mechanisms may underline septic kidney injury.

Although we did not directly confirm increased HuR expression in macrophages within LPS-injured kidneys, we observed that LPS stimulates HuR expression in cultured murine macrophages, similar to its effects on renal tubular cells. This suggests that injected LPS may upregulate HuR expression in both circulating and resident macrophages. HuR has been shown to mediate CD147 expression in macrophages, enhancing cytokine and chemokine production. Additionally, elevated CD147 levels may promote macrophage infiltration into LPS-injured kidneys through interactions with E-selectin ligands on the renal endothelium [25,32,36]. These findings propose a novel mechanism in which endotoxemia activates and recruits macrophages to kidney injury sites via the HuR-CD147 axis.

Together, our findings demonstrate that LPS-induced HuR expression in both immune and kidney cells drives tubulointerstitial inflammation and fibrosis. This study expands on our previous work [16,17], emphasizing the critical role of HuR-mediated post-transcriptional regulation in initiating and sustaining renal inflammation and fibrosis.

We previously identified KH3 as a potent HuR inhibitor through high-throughput screening. KH3 effectively inhibits HuR targets, ameliorating renal glomerulosclerosis, tubular interstitial fibrosis, and cardiac fibrosis [16–18,37]. However, KH3’s low solubility in buffer raised concerns regarding clinical applicability. To address this, we developed KH39, a more potent derivative with enhanced inhibitory activity, as demonstrated by a lower Ki value in fluorescence polarization assays [21]. While KH39’s solubility was not significantly improved, it has demonstrated efficacy in multiple *in vitro* and *in vivo* models, including tumor suppression via disruption of the HuR-mRNA interactions [21]. In this study, KH39 effectively blocked LPS-stimulated HuR expression in cultured macrophages, renal tubular epithelial cells, and a septic kidney injury model, highlighting the therapeutic potential of HuR inhibitors in inflammatory kidney diseases.

We also identified niclosamide (NCS), an FDA-approved anthelminthics drug, as a novel inhibitor of HuR cytoplasmic accumulation [22,23]. NCS has a well-established safety profile and multifunctional effects in drug repurposing screens, targeting pathways such as Wnt/*ß*-catenin, *m*TORC1, STAT3, and NF-kB [38–41]. Clinical trials are investigating NCS for cancer treatment, and one trial demonstrated its ability to reduce albuminuria in patients with diabetic nephropathy when combined with angiotensin-converting enzyme inhibitors [42]. However, the precise mechanism through which NCS exerts its effects remains unclear. In this study, NCS inhibited HuR expression and nucleocytoplasmic translocation in macrophages and kidney cells, suppressing HuR-targeted transcripts like CD147 in septic kidney disease. Although NCS does not directly bind to HuR like KH3 or KH39 [20–22], it may modulate HuR activity indirectly, potentially through effects on HuR phosphorylation or dimerization, which are crucial for HuR nucleocytoplasmic translocation and function [43]. These HuR-dependent and HuR-independent mechanisms likely contribute to NCS’s renoprotective effects in LPS-induced AKI.

This study has limitations that inform future research directions, including the need for longer-term observations of septic kidney injury in both male and female mice to assess the sustained effects and safety of NCS. Monitoring potential off-targeting effects will also be critical before clinical translation. Large cohort studies in patients will be necessary to validate these findings and assess clinical relevance. Nonetheless, our results highlight the therapeutic potential of NCS as a repurposed drug targeting HuR in progressive septic kidney disease.

**Figure 9:**
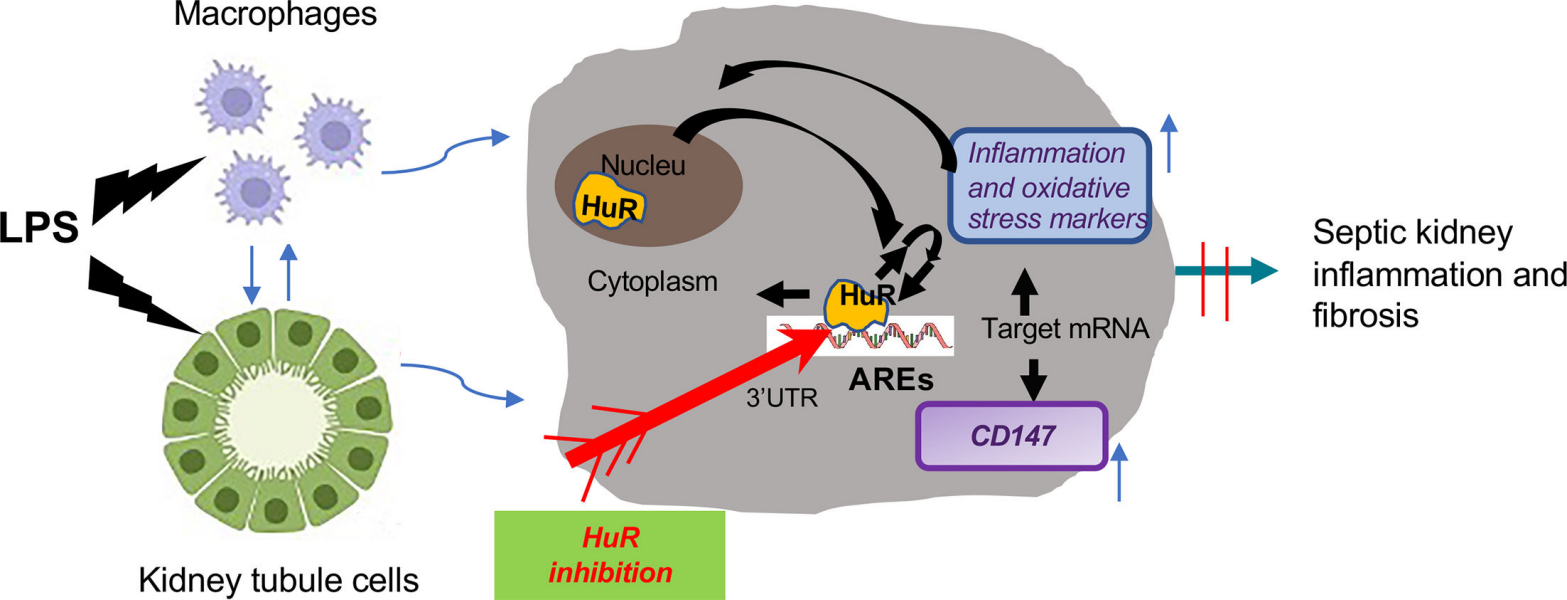
Schematic summary of the novel mechanism of septic kidney injury and the therapeutic potential. HuR serves as a predominant positive regulator of critical genes involved in inflammation and fibrosis. LPS exposure enhances HuR’s interaction with target transcripts, particularly CD147, in macrophages and renal tubular cells, leading to kidney injury. Inhibition of HuR may mitigate septic kidney injury by disrupting this pathological pathway.

In summary, our study reveals a novel mechanism by which HuR-mediated CD147 expression contributes to septic kidney injury and fibrosis (as illustrated in Figure 9). Targeting the HuR-CD147 axis represents a promising therapeutic strategy. By inhibiting HuR and CD147, NCS emerges as a potential repurposed drug with significant clinical promise for the treatment of progressive septic kidney disease or other kidney disorders.

**Clinical perspectives**

- Septic kidney injury, characterized by persistent inflammation, is a significant risk factor for developing CKD and currently lacks effective treatment.
- HuR serves as a key positive regulator of critical genes involved in inflammation and fibrosis. Exposure to LPS enhances the interaction between HuR and its target transcripts, particularly CD147, in macrophages and renal tubular cells, contributing to kidney injury.
- By inhibiting both HuR and CD147, niclosamide emerges as a potential repurposed drug and the first HuR inhibitor to be tested clinically for the treatment of progressive septic kidney disease or other forms of kidney diseases.

## Data Availability

The data supporting the findings of this study are available from the corresponding author upon reasonable request.

## Abbreviations

AKI: acute kidney injury
AREs: adenine- and uridine-rich elements
BUN: blood urea nitrogen
CKD: chronic kidney disease
CVD: cardiovascular disease
Col-III: type III collagen
ELAVL1: embryonic lethal abnormal vision-like protein
EMMPRIN: extracellular matrix metalloproteinase inducer
FN: fibronectin
HuR: Human antigen R
IF: immunofluorescent staining
LPS: lipopolysaccharide
NCS: niclosamide
NF-kB: nuclear transcription factor-kappa B
STAT3: signal transducer and activator of transcription 3
VSMCs: vascular smooth muscle cells
a-SMA: alpha-smooth muscle actin
mTORC1: mechanistic target of rapamycin complex 1

## References

[1] Peerapornratana S., Manrique-Caballero C.L., Gómez H., Kellum J.A (2019). Acute kidney injury from sepsis: current concepts, epidemiology, pathophysiology, prevention and treatment. Kidney Int..

[2] Kuwabara S., Goggins E., Okusa M.D (2022). The pathophysiology of sepsis-associated AKI. Clin. J. Am. Soc. Nephrol..

[3] Ma W.J., Cheng S., Campbell C., Wright A., Furneaux H (1996). Cloning and characterization of HuR, a ubiquitously expressed Elav-like protein. J. Biol. Chem..

[4] Mukherjee N., Corcoran D.L., Nusbaum J.D., Reid D.W., Georgiev S., Hafner M. (2011). Integrative regulatory mapping indicates that the RNA-binding protein HuR couples pre-mRNA processing and mRNA stability. Mol. Cell..

[5] Shang J., Zhao Z (2017). Emerging role of HuR in inflammatory response in kidney diseases. Acta Biochim. Biophys. Sin. (Shanghai).

[6] Kang M.J., Ryu B.K., Lee M.G., Han J., Lee J.H., Ha T.K. (2008). NF-kappaB activates transcription of the RNA-binding factor HuR, via PI3K-AKT signaling, to promote gastric tumorigenesis. Gastroenterology.

[7] Doller A., Akool E.S., Huwiler A., Müller R., Radeke H.H., Pfeilschifter J. (2008). Posttranslational modification of the AU-rich element binding protein HuR by protein kinase Cdelta elicits angiotensin II-induced stabilization and nuclear export of cyclooxygenase 2 mRNA. Mol. Cell. Biol..

[8] Bai D., Gao Q., Li C., Ge L., Gao Y., Wang H (2012). A conserved TGFβ1/HuR feedback circuit regulates the fibrogenic response in fibroblasts. Cell. Signal..

[9] Abdelmohsen K., Gorospe M (2010). Posttranscriptional regulation of cancer traits by HuR. Wiley Interdiscip. Rev. RNA.

[10] Shang J., Wan Q., Wang X., Duan Y., Wang Z., Wei X (2015). Identification of NOD2 as a novel target of RNA-binding protein HuR: evidence from NADPH oxidase-mediated HuR signaling in diabetic nephropathy. Free Radic. Biol. Med..

[11] Yu C., Xin W., Zhen J., Liu Y., Javed A., Wang R. (2015). Human antigen R mediated post-transcriptional regulation of epithelial-mesenchymal transition related genes in diabetic nephropathy. J. Diabetes.

[12] Doller A., Gauer S., Sobkowiak E., Geiger H., Pfeilschifter J., Eberhardt W (2009). Angiotensin II induces renal plasminogen activator inhibitor-1 and cyclooxygenase-2 expression post-transcriptionally via activation of the mRNA-stabilizing factor human-antigen R. Am. J. Pathol..

[13] Ceolotto G., De Kreutzenberg S.V., Cattelan A., Fabricio A.S., Squarcina E., Gion M. (2014). Sirtuin 1 stabilization by HuR represses TNF-α- and glucose-induced E-selectin release and endothelial cell adhesiveness in vitro: relevance to human metabolic syndrome. Clin. Sci. (Lond).

[14] Danilin S., Sourbier C., Thomas L., Lindner V., Rothhut S., Dormoy V. (2010). Role of the RNA-binding protein HuR in human renal cell carcinoma. Carcinogenesis.

[15] Feigerlová E., Battaglia-Hsu S.F (2017). Role of post-transcriptional regulation of mRNA stability in renal pathophysiology: focus on chronic kidney disease. FASEB J..

[16] Liu S., Huang Z., Tang A., Wu X., Aube J., Xu L. (2020). Inhibition of RNA-binding protein HuR reduces glomerulosclerosis in experimental nephritis. Clin. Sci. (Lond).

[17] Huang Z., Liu S., Tang A., Wu X., Aube J., Xu L. (2023). Targeting RNA-binding protein HuR to inhibit the progression of renal tubular fibrosis. J. Transl. Med..

[18] Green L.C., Anthony S.R., Slone S., Lanzillotta L., Nieman M.L., Wu X. (2019). Human antigen R as a therapeutic target in pathological cardiac hypertrophy. JCI Insight.

[19] Wu X., Lan L., Wilson D.M., Marquez R.T., Tsao W.C., Gao P. (2015). Identification and validation of novel small molecule disruptors of HuR-mRNA interaction. ACS Chem. Biol..

[20] Wu X., Gardashova G., Lan L., Han S., Zhong C., Marquez R.T (2020). Targeting the interaction between RNA-binding protein HuR and FOXQ1 suppresses breast cancer invasion and metastasis. Commun. Biol..

[21] Wu X., Ramesh R., Wang J., Zheng Y., Armaly A.M., Wei L. (2023). Small molecules targeting the RNA-Binding protein HuR Inhibit tumor growth in xenografts. J. Med. Chem..

[22] Yang Z., Zhang Q., Wu X., Hao S., Hao X., Jones E. (2023). Repurposing niclosamide as a novel Anti-SARS-CoV-2 drug by restricting entry protein CD147. Biomedicines.

[23] Zhang Q., Yang Z., Hao X., Dandreo L.J., He L., Zhang Y. (2023). Niclosamide improves cancer immunotherapy by modulating RNA-binding protein HuR-mediated PD-L1 signaling. Cell Biosci..

[24] Doi K., Leelahavanichkul A., Yuen P.S.T., Star R.A (2009). Animal models of sepsis and sepsis-induced kidney injury. J. Clin. Invest..

[25] Chen H., Zhu J., Liu Y., Dong Z., Liu H., Liu Y. (2015). Lipopolysaccharide induces chronic kidney injury and fibrosis through activation of mTOR signaling in macrophages. Am. J. Nephrol..

[26] Tian M., Tang L., Wu Y., Beddhu S., Huang Y (2018). Adiponectin attenuates kidney injury and fibrosis in deoxycorticosterone acetate-salt and angiotensin II-induced CKD mice. Am. J. Physiol. Renal Physiol..

[27] Tian M., Carroll L.S., Tang L., Uehara H., Westenfelder C., Ambati B.K. (2020). Systemic AAV10.COMP-Ang1 rescues renal glomeruli and pancreatic islets in type 2 diabetic mice. BMJ Open Diabetes Res. Care.

[28] Huang Z., Liu S., Tang A., Al-Rabadi L., Henkemeyer M., Mimche P.N. (2021). Key role for EphB2 receptor in kidney fibrosis. Clin. Sci. (Lond).

[29] Zhou G., Cheung A.K., Liu X., Huang Y (2014). Valsartan slows the progression of diabetic nephropathy in db/db mice via a reduction in podocyte injury, and renal oxidative stress and inflammation. Clin. Sci. (Lond).

[30] Zhou G., Wu J., Gu C., Wang B., Abel E.D., Cheung A.K. (2018). Prorenin independently causes hypertension and renal and cardiac fibrosis in cyp1a1-prorenin transgenic rats. Clin. Sci. (Lond).

[31] Kosugi T., Maeda K., Sato W., Maruyama S., Kadomatsu K (2015). CD147 (EMMPRIN/Basigin) in kidney diseases: from an inflammation and immune system viewpoint. Nephrol. Dial. Transplant..

[32] Bonomo I., Assoni G., La Pietra V., Canarutto G., Facen E., Donati G. (2023). HuR modulation counteracts lipopolysaccharide response in murine macrophages. Dis. Model. Mech..

[33] Guo J., Lei M., Cheng F., Liu Y., Zhou M., Zheng W. (2020). RNA-binding proteins tristetraprolin and human antigen R are novel modulators of podocyte injury in diabetic kidney disease. Cell Death Dis..

[34] Qu X., Wang C., Zhang J., Qie G., Zhou J (2014). The roles of CD147 and/or cyclophilin A in kidney diseases. Mediators Inflamm..

[35] Li L., Tang W., Wu X., Karnak D., Meng X., Thompson R. (2013). HAb18G/CD147 promotes pSTAT3-Mediated pancreatic cancer development via CD44s. Clin. Cancer Res..

[36] Kato N., Yuzawa Y., Kosugi T., Hobo A., Sato W., Miwa Y. (2009). The E-selectin ligand basigin/CD147 is responsible for neutrophil recruitment in renal ischemia/reperfusion. J. Am. Soc. Nephrol..

[37] Green L.C., Slone S., Anthony S.R., Guarnieri A.R., Parkins S., Shearer S.M (2023). HuR-dependent expression of Wisp1 is necessary for TGFβ-induced cardiac myofibroblast activity. J. Mol. Cell. Cardiol..

[38] Li Y., Li P.K., Roberts M.J., Arend R.C., Samant R.S., Buchsbaum D.J (2014). Multi-targeted therapy of cancer by niclosamide: A new application for an old drug. Cancer Lett..

[39] Li Z., Brecher M., Deng Y.Q., Zhang J., Sakamuru S., Liu B. (2017). Existing drugs as broad-spectrum and potent inhibitors for Zika virus by targeting NS2B-NS3 interaction. Cell Res..

[40] Chen W., Mook R.A., Premont R.T., Wang J (2018). Niclosamide: beyond an antihelminthic drug. Cell. Signal..

[41] Chang X., Zhen X., Liu J., Ren X., Hu Z., Zhou Z (2017). The antihelmenthic phosphate niclosamide impedes renal fibrosis by inhibiting homeodomain-interacting protein kinase 2 expression. Kidney Int..

[42] El-Fatatry B.M., El-Haggar S.M., Ibrahim O.M., Shalaby K.H (2023). Niclosamide from an anthelmintic drug to a promising adjuvant therapy for diabetic kidney disease: randomized clinical trial. Diabetol. Metab. Syndr..

[43] Pabis M., Popowicz G.M., Stehle R., Fernández-Ramos D., Asami S., Warner L. (2019). HuR biological function involves RRM3-mediated dimerization and RNA binding by all three RRMs. Nucleic Acids Res..

